# Adverse Childhood Experiences and Cardiovascular Risk among Young Adults: Findings from the 2019 Behavioral Risk Factor Surveillance System

**DOI:** 10.3390/ijerph191811710

**Published:** 2022-09-16

**Authors:** Dylan B. Jackson, Alexander Testa, Krista P. Woodward, Farah Qureshi, Kyle T. Ganson, Jason M. Nagata

**Affiliations:** 1 John Hopkins Bloomberg School of Public Health, Baltimore, MD 21205, USA; 2 Department of Management, Policy & Community Health, University of Texas Health Science Center at Houston, 1200 Pressler Street, Houston, TX 77030, USA; 3 Factor-Inwentash Faculty of Social Work, University of Toronto, Toronto, ON M5S 1A1, Canada; 4 Department of Pediatrics, University of California, San Francisco, 513 Parnassus Ave, San Francisco, CA 94143, USA

**Keywords:** cardiovascular, adverse childhood experiences, young adults, health

## Abstract

**Background:** Heart disease is the fourth leading cause of death for young adults aged 18–34 in the United States. Recent research suggests that adverse childhood experiences (ACEs) may shape cardiovascular health and its proximate antecedents. In the current study, we draw on a contemporary, national sample to examine the association between ACEs and cardiovascular health among young adults in the United States, as well as potential mediating pathways. **Methods:** The present study uses data from the 2019 Behavioral Risk Factor Surveillance System (BRFSS) to examine associations between ACEs and cardiovascular risk, as well as the role of cumulative disadvantage and poor mental health in these associations. **Results:** Findings indicate that young adults who have experienced a greater number of ACEs have a higher likelihood of having moderate to high cardiovascular risk compared to those who have zero or few reported ACEs. Moreover, both poor mental health and cumulative disadvantage explain a significant proportion of this association. **Conclusions:** The present findings suggest that young adulthood is an appropriate age for deploying prevention efforts related to cardiovascular risk, particularly for young adults reporting high levels of ACEs.

## 1. Introduction

Cardiovascular disease is a significant public health issue. Indeed, heart disease is the leading cause of death in the United States, accounting for approximately one in every four deaths [1,2]. Aside from the tremendous cost to human life, heart disease is associated with severe economic costs of approximately $363 billion annually, stemming from the cost of healthcare services, medicines, and lost productivity [2]. While the risk of cardiovascular disease increases alongside age [3,4], heart disease is the fourth leading cause of death for young adults aged 18–34 in the United States—behind only unintentional injury, suicide, and homicide [5]. Importantly, achieving ideal markers of cardiovascular health can be protective against developing cardiovascular disease. The American Heart Association (AHA) has developed Life’s Simple 7 as a means of identifying seven risk factors that people can improve through lifestyle changes to help achieve ideal cardiovascular health, including smoking, poor diet, physical activity, body weight, blood pressure, cholesterol, and blood glucose [6]. Studies have demonstrated that achieving ideal health status along these metrics of cardiovascular health is associated with a lower risk of cardiovascular disease [7,8] and mortality [9].

While much focus is on the proximate lifestyle characteristics that lead to poor cardiovascular outcomes, trajectories for poor cardiovascular health behaviors can often be set earlier in life [10]. Events and experiences occurring during childhood and adolescence can shape development and contribute to unhealthy behaviors and adverse health conditions that arise as individuals transition to adulthood [11]. Of notable importance are adverse childhood experiences (ACEs), which encompass experiences with abuse, neglect, and parental/household challenges [12]. An accumulating body of research details that ACEs are consequential experiences that are associated with deleterious health outcomes in adulthood [13,14]. Studies have also linked ACEs to individual cardiovascular risk factors later in life, including smoking [15], obesity [16], physical inactivity [12], diabetes mellitus [17], and high blood pressure [18]. In addition, a recent review found that adults with four or more ACEs compared to those with no ACEs have a more than two-fold higher risk of developing cardiovascular disease and a nearly two-fold higher risk of premature mortality [19].

A central theoretical rationale for the association between ACEs and cardiovascular risk factors later in life is the role of ACEs in undermining mental health [18,20]. The long-term influence of ACEs on stress and other adverse emotional and mental health responses is well established [21,22], and early adversity has even been shown to amplify the longitudinal associations between psychological stress and adult health outcomes [23]. Additionally, mental health difficulties undermine heart health and increase the frequency of behavioral health risks for cardiovascular disease [24,25]. In sum, adverse mental health states are a highly plausible pathway for explaining the link between ACEs and cardiovascular risk factors. Beyond these factors, it is also plausible that life-course indicators of cumulative disadvantage, such as low income and education, may also partly explain why ACEs tend to be associated with cardiovascular risk factors. To illustrate this, ACEs are known to increase the risk of cumulative disadvantage over time [26,27,28], and recent research also indicates higher rates of cardiovascular risk factors and disease among adults with lower levels of education and income [29,30]. Therefore, cumulative disadvantage also emerges as a plausible pathway linking early adversity to cardiovascular risk among adults.

While the evidence for a link between ACEs and cardiovascular risk factors is sound, as is the plausibility of multiple theoretical pathways, there are key gaps in the extant literature that the current study aims to address. First, the focus of existing literature is largely on cardiac events *after* the onset of heart disease, rather than focusing on an earlier life stage where improving cardiovascular health can have long-term benefits in reducing the odds of cardiovascular disease [19]. Indeed, scholars have recently speculated that early-life adversities can alter health and health behaviors in ways that harm cardiovascular health across the lifespan and ultimately lead to an increased risk of cardiovascular disease [31,32]. Second, and relatedly, existing research on the relationship between ACEs and cardiovascular health often focuses on select cardiovascular risk factors, rather than focusing on the clustering of risk within a single unified framework such as that recommended by the AHA [6]. Third, while studies have established a direct relationship between ACEs and certain components of cardiovascular risk, there has been limited examination of potential mediating factors. Notably, ACEs are connected to greater disadvantage in terms of educational attainment and income later in life [26,27,28], as well as poorer mental health [13,33], both of which may serve as critical mediating factors. To be sure, educational attainment [34], income [35], and mental health [24] are all associated with cardiovascular health. However, the degree to which these factors might mediate the association between ACEs and young adult cardiovascular health remains unexplored. This is an important oversight in the literature, considering that identifying relevant mediating pathways linking ACEs and cardiovascular health can inform preventive interventions [19,20]. Fourth, potential variability across key demographic characteristics is not always prioritized, which is critical to lay a foundation for targeted preventive intervention approaches given known disparities in cardiovascular health across race [36,37], sex [38,39], and urbanicity [40,41].

In the current study, we draw on contemporary and nationally representative data to examine the association between ACEs and cardiovascular health among young adults in the United States. Specifically, the aim of the current study is to investigate the following research questions:

1. Are ACEs associated with cardiovascular risk factors in young adulthood (ages 18–34)?
2. Is the relationship between ACEs and cardiovascular risk factors in young adulthood mediated by cumulative disadvantage (low education and income) and poor mental health?
3. Is the relationship between ACEs and cardiovascular risk factors consistent across participant race, sex, and urbanicity?

## 2. Materials and Methods

The 2019 Behavioral Risk Factor Surveillance System (BRFSS) data are employed in the present study. The BRFSS, which is funded by the Centers for Disease Control and Prevention (CDC), is a national system of ongoing health-related telephone surveys. Data are collected regarding adult residents’ (ages 18+) health-related risk behaviors, chronic health conditions, and use of preventive services. While the BRFSS was initially established in 1984 with only a subset of states, it now includes all 50 states as well as the District of Columbia (DC) and three U.S. territories. Beginning in the 2010s, cellular telephone use (in addition to landline use) was incorporated into the methodology (which includes Random Digit Dialing [RDD]) to increase coverage and validity of the data, while maintaining representativeness and updating the weighting methodology. It is the largest continuously conducted health survey system in the world, with more than 400,000 adults completing the survey each year. For the current study, we employ data from the 2019 survey; dates for the administration of the 2019 survey ranged from 2 January 2019 to 28 April 2020.

In the 2019 BRFSS, the states (as well as DC and U.S. territories) used a standardized core questionnaire, in addition to optional models and state-added questions. Thus, while all states participated, the viability of a given analysis is contingent on data availability based on whether states opted into certain modules or included their own set of questions. In the case of adverse childhood experiences (ACEs), only a subset of states included this optional module and ultimately reported results back to the CDC for inclusion in the 2019 BRFSS data. Considering our focus is on ACEs and cardiovascular risk among young adults (ages 18–34), we first restricted the sample to all states in which the optional ACEs module was included in their 2019 surveys and the results were reported back to the CDC (17 states); we then restricted the sample to participants ages 18–34, given our focus on young adults (for details on included states and sample sizes by state, see Appendix A Table A1). Only participants with valid data on all ACEs items were eligible for inclusion in the present study. Furthermore, given our focus on risk factors for future emergence of cardiovascular disease or a cardiac event, the small number of participants with a documented history of cardiovascular disease (i.e., heart attack, stroke, or coronary heart disease) are excluded from all analyses (*n* = 170). However, ancillary analyses including these participants produce substantively similar findings (for more details, see Appendix A Table A4). These exclusions resulted in a final analytic sample of 14,425 young adults. More recent surveys (e.g., 2020) were not included, as items on our outcome variable of interest were discontinued in 2020 [42]. The median weighted 2019 survey response rate was 49.4%, ranging from 37.3% to 73.1% across states [43]. For response rates pertaining to the states included in the current study, see Appendix A Table A1. As noted in prior research [44], “BRFSS prevalence rates [are] comparable to other national surveys which rely on self-reports.” The present study was exempt from Institutional Review Board approval, as it used de-identified data from a publicly available dataset.

### 2.1. Dependent Variable: Cardiovascular Risk

The outcome variable is a composite measure of cardiovascular risk. We developed the measure following the lead of an analysis of BRFSS data by Briggs and colleagues [45], who created a cardiovascular risk (i.e., poor cardiovascular health) score (0–7) derived from the seven lifestyle behaviors and health factors identified by the AHA as being critical to cardiovascular health, known as “Life’s Simple 7” [6]. Life’s Simple 7 is a very common approach to studying cardiovascular health, with poor scores portending higher cardiovascular risk as individuals age (e.g., increasing the risk of heart failure in the future) [8]. The 2019 BRFSS data include items that capture each of Life’s Simple 7; according to the AHA, poor cardiovascular health (or a poor Life’s Simple 7 score) is defined by the following seven factors: Current smoking, physical inactivity, obesity, poor diet, hypertension, diabetes, and high cholesterol. To calculate cardiovascular risk for the present study, we first dichotomized each of these self-reported indicators from BRFSS [45]. Our smoking measure included current smokers who have smoked more than 100 cigarettes in their lifetime. Our physical inactivity measure captured an individual’s failure to meet the CDC recommended guidelines of 150 min per week of moderate to vigorous activity [46]. Participants with a body mass index (BMI) of greater than or equal to 30 were classified as obese. Poor diet was defined as consuming fewer than five daily servings of fruit and vegetables, which is a reliable proxy of overall diet quality [47]. Participants also self-reported high blood pressure, blood sugar, and cholesterol and were thus coded as having hypertension, diabetes, and/or high cholesterol, respectively.

In the present study, we summed the 7 binary indicators for each participant to acquire a cardiovascular risk score ranging from 0 to 7. Given the age range of our sample (ages 18–34), this count measure is positively skewed, with relatively few respondents demonstrating all or even a majority of the indicators. Even so, we designated those exhibiting a majority of the indicators (i.e., four or more) as having “high” cardiovascular risk (10.33% of the sample), those with fewer, but still multiple indicators (i.e., 2 or 3 indicators) as “moderate” cardiovascular risk (59.45% of the sample), and those with only a single or no indicators as “low” cardiovascular risk (30.22%). Importantly, our coding scheme including three risk groups—low, moderate, and high—approximates approaches taken in prior cardiovascular health research focused on young adult samples [41,48].

### 2.2. Independent Variable: Adverse Childhood Experiences

The 2019 BRFSS included 11 items capturing retrospective reports of adverse childhood experiences occurring before the age of 18, all of which were included in the present study. Six of these items capture household challenges, including *household mental illness* (i.e., Did you live with anyone who was depressed, mentally ill, or suicidal?), *household alcohol use* (i.e., Did you live with anyone who was a problem drinker or alcoholic?), *household drug use* (i.e., Did you live with anyone who used illegal street drugs of who abused medications?), *household incarceration* (i.e., Did you live with anyone who served time or was sentenced to serve time in a prison, jail, or other correctional facility?), *parental separation/divorce* (i.e., Were you parents separated or divorced?), and *witness household violence* (i.e., Did your parents or adults in your home ever slap, hit, kick, punch, or beat each other up?). The remaining five items captured various forms of abuse, including *physical abuse* (i.e., Did a parent or adult in your home ever hit, beat, kick, or physically hurt you in any way?), *emotional abuse* (i.e., Did a parent or adult in your home ever swear at you, insult you, or put you down?), and *sexual abuse* (i.e., Did anyone at least five years older than you or an adult ever (1) touch you sexually?, (2) try to make you touch them sexually?, or (3) force you to have sex?).

Affirmative responses to any of the three sexual abuse items were coded as a 1 on the sexual abuse indicator, in line with prior research [49]. Given the high prevalence of ACEs in the sample, with nearly 3 in 4 participants reporting at least one ACE, we categorized ACEs using a coding strategy that mirrors prior BRFSS research [50] and research with adult samples demonstrating a similar ACEs distribution [22]. Specifically, we include the following categories: *zero ACEs* (26.35%), *one ACE* (21.92%), *two ACEs* (14.18%), *three or four ACEs* (18.18%), and *five or more ACEs* (19.37%).

### 2.3. Mediating Variables

***Cumulative Disadvantage*.** Our first category of mediators assesses cumulative disadvantage during young adulthood and includes (1) low education and (2) low income. Low education is a dichotomous variable in which participants who did not graduate high school or graduated high school/obtained a GED but did not attend college were assigned a value of 1 and those who attended college or obtained a higher education degree were assigned a value of 0. Low income is a five-category variable, with higher scores reflecting lower current personal income of the participant: $50,000 + (1), $35,000–<$50,000 (2), $25,000–>$35,000 (3), $15,000–<$25,000 (4), and <$15,000 (5).

***Poor Mental Health*.** Our second category of mediators assesses poor mental health during young adulthood and includes (1) depression diagnosis and (2) poor mental health days. Depression diagnosis is a dichotomous variable in which participants who have received a depression diagnosis (i.e., were told by a doctor or health professional that they have depression) are assigned a value of 1, whereas all others are assigned a value of 0. In the case of poor mental health days, respondents were asked, “Now thinking about your mental health, which includes stress, depression, and problems with emotions, for how many days during the past 30 days was your mental health NOT good?” We follow the lead of Bor and colleagues [51] in the present study and coded responses as no poor mental health days (0), 1–13 poor mental health days (1), and 14 or more poor mental health days (2).

### 2.4. Covariates

The following covariates were included in the multivariate models to minimize the likelihood of spurious results: Age (in years), sex (male = 1), race/ethnicity (Black, Hispanic, Asian/Pacific Islander, Native American/American Indian, Multiracial, and Other Race/Ethnicity, with White as the reference category), marital status (married = 1), and urbanicity (urban = 1).

### 2.5. Analytic Plan

The analysis proceeded as follows. First, we calculated descriptive statistics of all variables included in the study for the full sample of young adults and for subsamples stratified by low, moderate, and high cardiovascular risk. Next, we plotted the proportion of young adults exhibiting low vs. high cardiovascular risk by the number of ACEs. Third, we employed multinomial logistic regression to examine the association between ACEs and cardiovascular risk in this sample; we did so in a stepwise fashion, first with baseline covariates included and then with cumulative disadvantage and poor mental health mediators added. In ancillary analyses, we also examined the consistency of results across individual ACEs and ACE categories and explored these models stratified by respondent sex, race, age, and urbanicity. Finally, we examined the role of the cumulative disadvantage and poor mental health mediators in the link between ACEs and cardiovascular risk using the Karlson–Holm–Breen (KHB) method [52]. There is ample precedent for using the KHB method to simultaneously examine multiple correlated mediators to predict categorical or non-linear outcomes [53,54]. We chose the KHB method for this analysis for two reasons. First, coefficients across nested nonlinear models cannot be directly compared because of a rescaling of the model that occurs after additional variables are added. The KHB corrects for this rescaling and provides an estimate of how much each variable mediates the association between the independent variable (ACEs) and dependent variable (cardiovascular risk). Second, because we are simultaneously considering multiple, correlated mediators, the KHB method provides the benefit of (1) decomposing the mediating effects of each of these individual variables and (2) calculating whether the change in the focal independent variable across models is greater than expected by change. All analyses were conducted in STATA 17.1 using multiply imputed data (chained equations, 20 imputations). Estimates are weighted to be representative of the populations in eligible states.

## 3. Results

We began by calculating descriptive statistics for the full analytic sample (*n* = 14,255) as well as subsamples with low (*n* = 4308), moderate (*n* = 8474), and high (*n* = 1473) cardiovascular risk. The findings are displayed in Table 1. In the full sample, ACEs were quite common, with nearly three in four participants reporting at least one ACE and almost one in five reporting five or more ACEs. The sample was, on average, 26.42 years old, and 51.23% were male. The sample was 67.65% white, 11.95% Black, 12.54% Hispanic, 2.80% Asian/pacific islander, 1.37% Native American/American Indian, 2.88% multiracial, and 5.66% other race/ethnicity. Only 28.84% were married, and 87.63% lived in urban areas. Additionally, 37.03% were categorized as having low education (i.e., did not attend or graduate college) and 23.11% had received a depression diagnosis.

The findings also indicate that the greatest proportion of respondents with the highest number of ACEs appear in the high cardiovascular risk group (relative to moderate and low). For instance, while nearly 30% of those with high cardiovascular risk report five or more ACEs, only 16% of those with low cardiovascular risk do. Inversely, nearly one in three young adults with low cardiovascular risk report no ACEs, while only 16.31% of young adults with high cardiovascular risk report no ACEs. Higher cardiovascular risk was also associated with increased age, was slightly higher among Black participants yet lower among Asian/pacific islander participants, and slightly lower among urban participants. In terms of the mediators, those with higher cardiovascular risk have a much higher prevalence of low education and depression diagnosis, and also scored higher on the low-income measure and poor mental health days.

**Figure 1:**
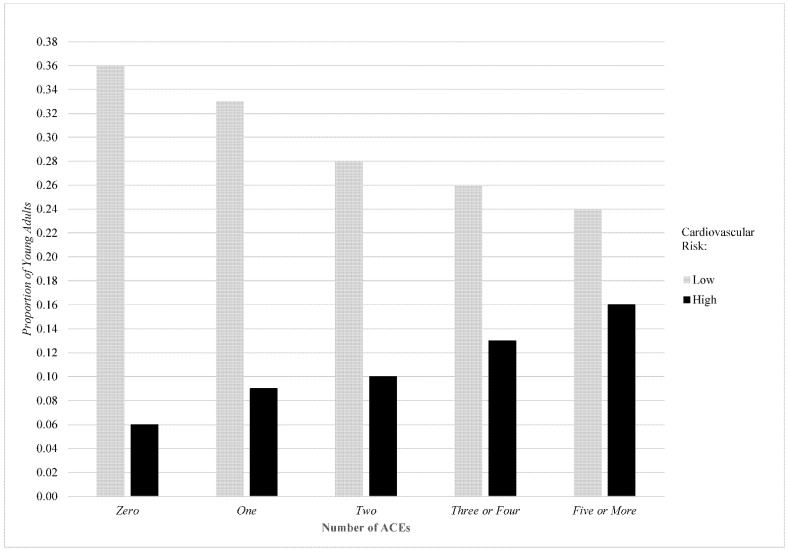
Proportion of young adults exhibiting low vs. high cardiovascular risk by number of ACEs.

Next, we plotted the proportion of young adults exhibiting low vs. high cardiovascular risk by the number of ACEs (see Figure 1). The findings indicate that while only 6% of respondents with zero ACEs reported high cardiovascular risk, 16% of those with five or more ACEs did. Additionally, the figure reveals a gradual uptick in the percentage of respondents with high cardiovascular risk with the addition of exposure to more ACEs.

Next, we estimate multivariable models of the association between ACEs and young adult cardiovascular risk using multinomial logistic regression. The findings are displayed in Table 2. The results from model 1 indicate that, even after adjusting for baseline covariates, the association between ACEs and increased cardiovascular risk among young adults holds. For instance, experiencing five or more ACEs is associated with a 62% increase in the risk of moderate cardiovascular risk (relative to low cardiovascular risk; *p* < 0.01) and a 339% increase in the risk of high cardiovascular risk (relative to low cardiovascular risk; *p* < 0.01). These results indicate a sizeable increase in the risk of high vs. moderate cardiovascular risk in the presence of accumulating ACEs. Notably, other predictors of high cardiovascular risk in the models are age (RRR = 1.15; CI = 1.13–1.18), male gender (RRR = 1.44; CI = 1.21–1.70), Black (RRR = 1.34; CI = 1.03–1.74) and Asian/Pacific Islander (RRR = 0.37; CI = 0.18–0.75) ethnicities, and urbanicity (RRR = 0.61; CI = 0.48–0.77). Notably, the link between ACEs and cardiovascular risk retained their significance across subsamples distinguished by sex, race, age, and urbanicity and when individual ACEs and ACE categories were examined (for more details, see Appendix A Table A2). Next, in model 2, we added the mediators to examine the initial attenuation of the ACEs coefficients. As expected, all ACEs coefficients were meaningfully reduced, but remained consistently statistically significant. Additionally, each of the four mediators of interest—low education, low income, depression diagnosis, and poor mental health days—was associated with a significantly higher relative risk of both moderate and high cardiovascular risk (relative to low cardiovascular risk). For instance, low education was associated with a 93% increase in the risk of high cardiovascular risk (relative to low), while a depression diagnosis was associated with a 109% increase in the risk of high cardiovascular risk (relative to low).

Finally, we examined the extent to which our indicators of cumulative disadvantage and poor mental health explained associations between ACEs and cardiovascular risk. Ancillary analyses first revealed expected, positive associations between cumulative ACEs and each of the proposed mediators, particularly as the number of ACEs increased (see Appendix A Table A5 and Table A6). The findings of the KHB analysis, however, are displayed in Table 3. Overall, there is substantial evidence that these factors explain a sizable and statistically significant portion of the association between ACEs and cardiovascular risk, especially when examining associations between five or more ACEs and moderate or high cardiovascular risk (49.77–50.67%). In the case of five or more ACEs, low education explained 7.76–8.44% of the association, low income explained 7.19–9.01% of the association, a depression diagnosis explained 16.03–21.07% of the association, and poor mental health days explained 13.97–16.97% of the association. In total, approximately 50% of the association between five or more ACEs and both moderate and high cardiovascular risk (relative to low) is explained collectively by these four mediators. Total mediation is somewhat less pronounced as ACEs are reduced, with low income in particular becoming non-significant as a mediator in the case of only one or two ACEs.

## 4. Discussion

Identifying cardiovascular risk in young adults is a primary public health concern given its impact on longevity and quality of life, as well as its economic burden in the United States [1,2]. Previous research has linked ACEs to cardiovascular disease and poor cardiovascular health [12,19,20]. Even so, the primary focus of this research has been on the association of ACEs and cardiovascular *disease* in *older* adults as opposed to cardiovascular *risk* in *young* adults, leaving noteworthy gaps in our understanding of cardiovascular disease risk mechanisms, markers, and overall burden [7]. Little attention has been paid to *how* the constellation of childhood adversities and cumulative disadvantage might meaningfully contribute to a young person’s risk profile as it relates to cardiovascular health. The current study used the 2019 BRFSS data to investigate whether the presence of ACEs was associated with worse cardiovascular risk in young adulthood (18–34 years old), and whether this relationship is mediated by cumulative disadvantage and poor mental health. Findings have the potential to inform clinicians’ protocol to include screening for ACEs in routine clinical cardiovascular encounters with young adults, as well as strategies to help prevent the onset of CVD later in life [19].

The primary results showed that there was a dose–response relationship between the number of reported ACEs and high cardiovascular risk, as compared to low or moderate cardiovascular risk. This finding is consistent with, yet expands upon, prior research [19], while also underscoring the deep impact of cumulative adversity and trauma experienced in childhood and its health manifestations in young adulthood. Additionally, these findings provide the first evidence that both cumulative disadvantage and poor mental health partially explained associations between ACEs and cardiovascular risk, yet there is little evidence of demographic variation in the association between ACEs and cardiovascular risk. The results allude to a potential point of intervention early in the life course among diverse, ACE-exposed youth to divert them from unhealthy pathways toward cardiovascular risk, and possibly eventual cardiovascular disease, via mental health support interventions and educational and economic opportunities.

Taken altogether, young adults who have experienced a greater number of ACEs have a higher likelihood of having moderate to high cardiovascular risk compared to those who have zero or few reported ACEs. This is an important distinction from the development of cardiovascular disease, as it provides time for prevention efforts to curb cardiovascular risk behaviors and disease onset. By examining ACEs and cumulative disadvantage as risk factors for cardiovascular risk, practitioners can more precisely target patients for intervention at an earlier age, thereby delaying or completely eliminating the onset of cardiovascular disease [11,12].

To date, ACE screenings are uncommonly incorporated in primary care protocols, let alone cardiovascular clinical prevention practices, leaving the field with a dearth of implementation guidance and recommendations [55,56]. Screening for ACEs and other health risks is especially germane to the present study’s target population (i.e., young adults), given low rates of primary care utilization and increased rates of high-risk behaviors [57,58]. Recent data show that young adults generally have a significant cardiovascular health risk resulting from heightened stress, poor diet, lack of sleep, decreased physical activity, and increased tobacco, alcohol, and drug use [59]. Compounding these known health risks is the deficient “transition of care” process from pediatric to adult medicine for young adults [57,60]. As a result, many young adults do not receive age-appropriate screening or anticipatory guidance promoting healthy behaviors that may prevent chronic conditions, such as cardiovascular disease [57,61].

Research has also shown that the majority of young adults do not accurately perceive their own risk for developing heart disease, which may also delay health care intervention [59,62]. Offering hope, previous studies found that many young adults are willing to change their lifestyle if their behaviors have been identified as risk factors for CVD and other chronic diseases by a clinician [59]. Therefore, primary care plays a major role in educating patients and changing negative health behaviors to minimize impacts earlier and reduce chronic disease onset. Future research is needed to better understand how transitional care can be more responsive to the needs of young adults, especially those who have a history of ACEs, and provide necessary prevention measures to mitigate CVD risk.

In addition to continuity in care, findings from the current study also increase the importance of trauma-informed, “whole person” care when treating cardiovascular risk in young adults. Cardiovascular risk is not simply a product of genetics or physical status; rather, these disparities are rooted in social determinants of health, including, but not limited to, adverse childhood experiences [19,32]. It is important to recognize the socioenvironmental risk factors contributing to cardiovascular risk, ensuring that interventions are not overly medicalized, but take the whole person into account, especially past traumas [63]. Research has shown some efficacy with trauma-informed primary care that helps personalize care and meet the complex needs of patients with trauma histories [19,64,65]. Additionally, some existing research intimates that trauma-informed care standards, such as conducting ACEs screenings or integrating education on the harmful effects of ACE exposure, may reduce the likelihood of future cardiovascular events [19,32]. By adopting a “whole person health” philosophy for primary care visits with young adults, practitioners are better able to assess cardiovascular risk and provide interventions beyond traditional pathophysiological treatments—such as cognitive behavior therapy, mindfulness, and/or psychodynamic therapy—that may treat the root of the problem [14,63]. Future research into post-ACE interventions and their effects on cardiovascular disease and risk is needed to expand services, particularly to vulnerable young people [19].

Finally, we should also note that there is much that can be achieved outside of clinical or health care settings to address ACEs early in the life course and thereby improve long-term cardiovascular health. For instance, research on community interventions for cardiovascular disease points to programs that modify cardiovascular risk factors—including ACEs—“in entire communities rather than targeting only high-risk individuals in health care settings” [66]. We agree that this approach is wise and has an important role to play in curtailing cardiovascular disease in the population. A recent systematic review revealed that most research shows that community-based interventions significantly improve knowledge related to cardiovascular risk factors and disease [67], which can help empower communities to make heart-healthy lifestyle choices. Even so, access to resources to offset the ACEs burden, and the risk factors incurred for cardiovascular disease among individuals in under-resourced, low-income communities presents a continual challenge [68]. Despite these barriers, efforts have been made to expand key resources—such as trauma-informed cognitive-behavioral therapy—into low-income communities [68], such as the Elijah Cummings Healing City Baltimore Act in Baltimore City, MD [69]. Furthermore, in low-income settings, it may be that hybrid models targeting children and their families with “options to engage through communities, schools, and the family unit” may be promising [70]. Ultimately, while cardiovascular health education is critical, systemic change that promotes equitable access to health resources that can stem the tide of cardiovascular disease remains a priority.

This study was not without limitations, including the cross-sectional nature of the BFRSS data source, which precludes definitive causal conclusions and limits our understanding of long-term disease risk progression and pathophysiology. Additionally, the BRFSS relies on self-reporting of sensitive information including adverse childhood experiences and disadvantages, which are subject to recall error and social desirability bias. Due to sample exclusion criteria (e.g., data availability by state, age range, and pre-existing cardiovascular conditions), the generalizability of the results may also be limited. Lastly, the traditional ACEs survey used for the BFRSS is missing other important childhood adversities including neglect, racism, police violence, and others that are currently being integrated into novel ACE frameworks [71]. Therefore, researchers and practitioners should consider additional traumatic experiences beyond the interpersonal level when studying and assessing cardiovascular health.

## 5. Conclusions

The present findings suggest that young adulthood is an appropriate age for deploying prevention efforts related to cardiovascular risk, particularly for young adults reporting high levels of ACEs. These findings confirm the notion that the social environment, starting at an early age, has a significant impact on our cardiovascular health and wellness [13,14,19]. Future cardiovascular health prevention efforts for adolescents and young adults should consider ACEs and mental health screenings, given their deleterious impacts. In addition, young adults who have high social-related health needs, including a low income or educational background, should also be considered at elevated risk for cardiovascular risk. Given the high rates of ACEs and mental health challenges, universal screening might be considered for population-level prevention of cardiovascular risk. Even so, the differential exposures to cumulative disadvantage and ACEs resulting in moderate to high cardiovascular risk necessitate different prevention plans tailored to each individual. By screening for ACEs and mental health conditions during young adulthood, practitioners can more accurately assess cardiovascular risk earlier and reduce the individual and societal burden associated with poor cardiovascular health. Future research in this space could explore how positive childhood experiences (PCEs) may buffer harmful impacts and how ACE screening and evidence-based interventions are able to reduce cardiovascular risk among young adults.

## Figures and Tables

**Table 1:** Descriptive statistics (*n* = 14,255).

		Cardiovascular Risk		
	Full Sample (*n* = 14,255)	Low (*n* = 4308)	Moderate (*n* = 8474)	High (*n* = 1473)
Variables	Mean (SD) or %	Mean (SD) or %	Mean (SD) or %	Mean (SD) or %
***ACEs***				
Zero	26.35%	31.20%	25.46%	16.31%
One	21.92%	23.59%	21.45%	18.87%
Two	14.18%	12.94%	14.85%	13.48%
Three or Four	18.18%	16.19%	18.57%	22.09%
Five or More	19.37%	16.09%	19.67%	29.26%
***Covariates***				
Age	26.42 (4.92)	25.62 (5.03)	26.48 (4.85)	28.50 (4.32)
Male	51.23%	50.75%	51.19%	52.47%
White	67.65%	68.90%	66.87%	67.52%
Black	11.95%	10.12%	12.54%	14.00%
Hispanic	12.54%	12.27%	12.96%	11.24%
Asian/Pacific Islander	2.80%	3.67%	2.70%	0.99%
Native American/American Indian	1.37%	1.50%	1.23%	2.04%
Multiracial	2.88%	2.74%	2.96%	3.02%
Other Race/Ethnicity	5.66%	0.81%	0.75%	1.18%
Married	28.84%	29.10%	28.53%	29.52%
Urbanicity	87.63%	89.97%	87.10%	83.76%
***Mediators***				
*Cumulative Disadvantage*				
Low Education	37.03%	32.33%	37.72%	47.73%
Low Income	2.31 (1.40)	1.90 (1.32)	2.08 (1.38)	2.42 (1.48)
*Poor Mental Health*				
Depression Diagnosis	23.11%	17.69%	23.25%	40.89%
Poor Mental Health Days	0.73 (0.75)	0.66 (0.73)	0.75 (0.77)	0.98 (0.83)

Participants with a history of cardiovascular disease (i.e., heart attack, stroke, or coronary heart disease) are excluded from all analyses (*n* = 170). Estimates represent results from the BRFSS 2019 survey, participants ages 18–34 years.

**Table 2:** Multinomial logistic regression models of the association between ACEs and cardiovascular risk among young adults aged 18–34 years (*n* = 14,255).

	Model 1		Model 2	
Variables	*Moderate* RRR CI	*High* RRR CI	*Moderate* RRR CI	*High* RRR CI
***ACEs***				
One	1.19 * 1.03–1.38	1.79 ** 1.37–2.34	1.16 * 1.00–1.34	1.59 ** 1.21–2.09
Two	1.55 ** 1.32–1.83	2.47 ** 1.85–3.30	1.48 ** 1.25–1.75	1.99 ** 1.48–2.68
Three or Four	1.40 ** 1.20–1.64	3.24 ** 2.48–4.22	1.28 ** 1.09–1.50	2.26 ** 1.72–2.95
Five or More	1.62 ** 1.38–1.91	4.39 ** 3.41–5.64	1.37 ** 1.16–1.62	2.31 ** 1.77–3.01
***Covariates***				
Age	1.04 ** 1.03–1.05	1.15 ** 1.13–1.18	1.05 ** 1.04–1.06	1.16 ** 1.14–1.18
Male	1.14 * 1.03–1.27	1.44 ** 1.21–1.70	1.19 ** 1.07–1.33	1.69 ** 1.42–2.01
Black	1.20 * 1.02–1.42	1.34 * 1.03–1.74	1.17 0.99–1.39	1.38 * 1.06–1.80
Hispanic	1.12 0.93–1.36	1.26 0.92–1.73	1.08 0.89–1.31	1.19 0.86–1.64
Asian/Pacific Islander	0.89 0.67–1.19	0.37 ** 0.18–0.75	0.91 0.68–1.22	0.44 * 0.22–0.90
Native American/American Indian	0.71 0.42–1.18	0.94 0.47–1.88	0.66 0.40–1.11	0.72 0.37–1.41
Multiracial	1.02 0.76–1.37	1.00 0.61–1.67	0.97 0.72–1.30	0.88 0.52–1.48
Other Race/Ethnicity	1.08 0.62–1.88	1.39 0.61–3.17	1.10 0.64–1.90	1.57 0.69–3.58
Married	0.81 ** 0.71–0.92	0.61 0.50–0.74	0.87 * 0.76–0.99	0.79 * 0.65–0.97
Urbanicity	0.80 ** 0.68–0.93	0.61 ** 0.48–0.77	0.81 * 0.69–0.95	0.65 ** 0.51–0.83
***Mediators***				
*Cumulative Disadvantage*				
Low Education	-	-	1.24 ** 1.11–1.39	1.93 ** 1.61–2.31
Low Income	-	-	1.08 ** 1.03–1.12	1.15 ** 1.08–1.23
*Poor Mental Health*				
Depression Diagnosis	-	-	1.16 * 1.00–1.34	2.09 ** 1.70–2.59
Poor Mental Health Days	-	-	1.12 ** 1.04–1.21	1.43 ** 1.27–1.62

*Note:* ** *p* < 0.01; * *p* < 0.05. RRR = Relative Risk Ratio. CI = Confidence Interval. Reference category for Cardiovascular Risk is “low”. Reference category for categorical measures of ACEs is “zero”. Reference category for Race/Ethnicity is “White”. Estimates are weighted to represent the U.S. population in eligible states of young adults aged 18 to 34 years. Participants with a history of cardiovascular disease (i.e., heart attack, stroke, or coronary heart disease) are excluded from all analyses (*n* = 170). Models adjust for state-specific effects. Estimates represent results from the BRFSS 2019 survey.

**Table 3:** KHB test of mediators between ACE exposure and young adult cardiovascular risk.

	Moderate Cardiovascular Risk (*Ref: Low Cardiovascular Risk*)							
*Mediators*	*One ACE*		*Two ACEs*		*Three or Four ACEs*		*Five or More ACEs*	
*Cumulative Disadvantage*	% Reduction	*z*-score	% Reduction	*z*-score	% Reduction	*z*-score	% Reduction	*z*-score
Low Education	6.87%	2.57 **	2.61%	2.65 **	3.80%	3.53 **	7.76%	4.63 **
Low Income	2.97%	0.92	−0.32	−0.28	4.96%	3.85 **	9.01%	5.65 **
***Poor Mental Health***								
Depression Diagnosis	8.65%	3.15 **	7.34%	3.56 **	10.99%	3.66 **	16.03%	3.70 **
Poor Mental Health Days	14.04%	3.56**	9.33%	3.84 **	12.84%	3.93 **	16.97%	3.97 **
*Total*	**32.53%**	**-**	**18.96%**	**-**	**32.59%**	**-**	**49.77%**	**-**
	**High Cardiovascular Risk** (*Ref: Low Cardiovascular Risk*)							
***Mediators***	*One ACE*		*Two ACEs*		*Three or Four ACEs*		*Five or More ACEs*	
***Cumulative Disadvantage***	% Reduction	*z*-score	% Reduction	*z*-score	% Reduction	*z*-score	% Reduction	*z*-score
Low Education	3.00%	1.29	3.51%	2.01 *	3.64%	3.05 **	8.44%	6.68 **
Low Income	0.01%	0.01	−0.92%	−0.54	3.04%	2.54 **	7.19%	6.06 **
***Poor Mental Health***								
Depression Diagnosis	10.66%	4.84 **	13.29%	6.24 **	16.21%	7.86 **	21.07%	8.71 **
Poor Mental Health Days	7.52%	3.92 **	12.12%	5.29 **	10.70%	5.62 **	13.97%	5.93 **
*Total*	**21.19%**	**-**	**28.00%**	**-**	**33.59%**	**-**	**50.67%**	**-**

** *p* < 0.01, * *p* < 0.05.

## Data Availability

Further information on the BRFSS data and access to the data can be found at: https://www.cdc.gov/brfss/.

## Appendix A

**Table A1:** List of states in analytic sample (BRFSS 2019 Survey, young adults aged 18–34).

State	Sample Size (N)	Response Rate
Alabama	715	45.9%
Delaware	481	38.2%
Florida	1665	44.3%
Indiana	926	46.2%
Iowa	1369	58.7%
Michigan	1460	51.5%
Mississippi	639	57.4%
Missouri	849	56.2%
New Mexico	684	52.2%
North Dakota	602	60.8%
Pennsylvania	984	46.6%
Rhode Island	546	43.6%
South Carolina	803	54.6%
Tennessee	656	42.0%
Virginia	1100	43.4%
West Virginia	448	49.6%
Wisconsin	498	53.3%
**Total/Average**	**14,425**	**49.68%**

Note: States were eligible for inclusion in the present study if they included all adverse childhood experiences (ACEs) items in their 2019 surveys. Note that states including the ACE module in their 2019 surveys did so using their own resources and are not required to report ACE data back to the Center of Disease Control and Prevention (CDC) Behavioral Risk Factor Surveillance System (BRFSS). Thus, states are represented only if they provided ACEs data to CDC BRFSS.

**Table A2:** Multinomial logistic regression models of the association between ACEs and cardiovascular risk among young adults stratified by sex, race, age, and urbanicity.

Variables	*Moderate* RRR (CI)	*High* RRR (CI)	*Moderate* RRR (CI)	*High* RRR (CI)
	Male (*n* = 7303)		Female (*n* = 6952)	
***ACEs***				
One	1.24 * (1.02–1.51)	2.23 ** (1.56–3.20)	1.15 (0.93–1.42)	1.35 (1.37–2.34)
Two	1.39 ** (1.11–1.74)	2.07 ** (1.41–3.03)	1.81 ** (1.42–2.30)	3.08 ** (1.97–4.80)
Three or Four	1.41 ** (1.14–1.75)	3.36 ** (2.34–4.82)	1.40 ** (1.12–1.76)	3.13 ** (2.11–4.63)
Five or More	1.55 ** (1.23–1.95)	4.24 ** (2.97–6.05)	1.70 ** (1.36–2.12)	4.48 ** (3.12–6.44)
	White (*n* = 9644)		Non-White (*n* = 4611)	
***ACEs***				
One	1.30 ** (1.10–1.53)	1.80 ** (1.33–2.44)	1.00 (0.76–1.33)	1.75 * (1.04–2.94)
Two	1.59 ** (1.31–1.94)	2.80 ** (1.98–3.97)	1.45 * (1.07–1.97)	1.85 * (1.10–3.12)
Three or Four	1.47 ** (1.23–1.76)	3.02 ** (2.20–4.14)	1.27 (0.94–1.73)	3.68 ** (2.27–5.96)
Five or More	1.75 ** (1.46–2.11)	4.53 ** (3.34–6.12)	1.38 * (1.00–1.90)	4.04 ** (2.55–6.39)
	18–26 Years Old (*n* = 6950)		27–34 Years Old (*n* = 7305)	
***ACEs***				
One	1.22 (0.99–1.49)	1.61 (0.98–2.66)	1.19 (0.97–1.47)	1.89 ** (1.39–2.58)
Two	1.42 ** (1.13–1.78)	1.85 * (1.13–3.03)	1.75 ** (1.37–2.24)	3.01 ** (2.08–4.36)
Three or Four	1.40 ** (1.13–1.74)	2.82 ** (1.81–4.40)	1.42 ** (1.13–1.77)	3.48 ** (2.49–4.86)
Five or More	1.54 ** (1.24–1.92)	3.41 ** (2.21–5.26)	1.72 ** (1.35–2.19)	5.04 ** (3.67–6.92)
	Urban/Suburban County (*n* = 12,492)		Rural County (*n* = 1763)	
***ACEs***				
One	1.16 (0.99–1.36)	1.81 ** (1.35–2.42)	1.53 * (1.04–2.27)	1.87 (0.99–3.52)
Two	1.53 ** (1.29–1.82)	2.60 ** (1.90–3.55)	1.74 * (1.04–2.92)	1.85 (0.81–4.23)
Three or Four	1.38 ** (1.17–1.62)	3.41 ** (2.57–4.54)	1.71 * (1.08–2.68)	2.15 * (1.08–4.31)
Five or More	1.57 ** (1.33–1.86)	4.44 ** (3.38–5.83)	2.57 ** (1.59–4.13)	5.04 ** (2.57–9.89)

Note: ** *p* < 0.01; * *p* < 0.05. RRR = Relative Risk Ratio. CI = Confidence Interval. Reference category for Cardiovascular Risk is “low”. Reference category for categorical measures of ACEs is “zero”. All models adjust for age, sex, race/ethnicity, marital status, and urbanicity. Models also adjust for state-specific effects. Estimates are weighted to represent the U.S. population in eligible states of young adults ages 18 to 34 years. Participants with a history of cardiovascular disease (i.e., heart attack, stroke, or coronary heart disease) are excluded from all analyses (*n* = 170). Estimates represent results from the BRFSS 2019 survey.

**Table A3:** Multinomial logistic regression models of the association between individual ACEs and cardiovascular risk among young adults (*n* = 14,255).

Variables	*Moderate* RRR CI	*High* RRR CI
***ACEs***		
Family Member Mental Illness	1.21 ** (1.08–1.36)	1.98 ** (1.66–2.35)
Family Member Alcoholic	1.22 ** (1.09–1.38)	1.73 ** (1.45–2.07)
Family Member Used Illegal Drugs	1.35 ** (1.18–1.55)	2.16 ** (1.78–2.62)
Family Member Incarcerated	1.27 ** (1.10–1.47)	1.92 ** (1.54–2.39)
Parents Divorced	1.22 ** (1.10–1.36)	1.79 ** (1.51–2.11)
Physical Violence Between Parents	1.37 ** (1.20–1.56)	1.79 ** (1.47–2.17)
Parent Physically Abused Child	1.34 ** (1.18–1.52)	1.98 ** (1.64–2.38)
Parent Verbally Abused Child	1.18 ** (1.06–1.31)	1.90 ** (1.60–2.25)
Child Sexual Abuse	1.29 ** (1.10–1.53)	2.40 ** (1.91–3.01)

Note: ** *p* < 0.01. RRR = Relative Risk Ratio. CI = Confidence Interval. Reference category for Cardiovascular Risk is “low”. All models adjust for age, sex, race/ethnicity, marital status, and urbanicity. Models also adjust for state-specific effects. Estimates are weighted to represent the U.S. population in eligible states of young adults ages 18 to 34 years. Participants with a history of cardiovascular disease (i.e., heart attack, stroke, or coronary heart disease) are excluded from all analyses (*n* = 170). Estimates represent results from the BRFSS 2019 survey.

**Table A4:** Multinomial logistic regression models of the association between ACEs and cardiovascular risk among young adults aged 18–34 years, including those with a history of cardiovascular disease (*n* = 14,425).

Variables	*Moderate* RRR CI	*High* RRR CI
***ACEs***		
One	1.19 * (1.03–1.37)	1.76 ** (1.35–2.29)
Two	1.56 ** (1.33–1.84)	2.46 ** (1.84–3.27)
Three or Four	1.40 ** (1.20–1.64)	3.18 ** (2.45–4.13)
Five or More	1.63 ** (1.39–1.92)	4.25 ** (3.31–5.45)
***Covariates***		
Age	1.04 ** (1.03–1.05)	1.15 ** (1.13–1.18)
Male	1.15 ** (1.03–1.27)	1.44 ** (1.22–1.70)
Black	1.20 * (1.02–1.41)	1.31 * (1.01–1.70)
Hispanic	1.13 (0.93–1.36)	1.27 (0.93–1.73)
Asian/Pacific Islander	0.87 (0.65–1.16)	0.35 ** (0.17–0.71)
Native American/American Indian	0.70 (0.42–1.16)	0.98 (0.50–1.92)
Multiracial	1.01 (0.76–1.35)	1.11 (0.69–1.79)
Other Race/Ethnicity	1.09 (0.63–1.89)	1.56 (0.70–3.46)
Married	0.81 ** (0.71–0.92)	0.61 (0.50–0.74)
Urbanicity	0.79 ** (0.67–0.93)	0.62 ** (0.49–0.78)
Cardiovascular Disease	1.45 (0.79–2.64)	3.45 ** (1.73–6.86)

Note: ** *p* < 0.01; * *p* < 0.05. RRR = Relative Risk Ratio. CI = Confidence Interval. Reference category for Cardiovascular Risk is “low”. Reference category for categorical measures of ACEs is “zero”. Reference category for Race/Ethnicity is “White”. Estimates are weighted to represent the U.S. population in eligible states of young adults ages 18 to 34 years. Models adjust for state-specific effects. Estimates represent results from the BRFSS 2019 survey.

**Table A5:** Association between adverse childhood experiences and indicators of cumulative disadvantage among young adults aged 18–34 (*n* = 14,255).

	*Low Education*	*Low Income*
Variables	OR (CI)	B/Beta (SE)
***ACEs***		
One	1.26 ** (1.09–1.46)	0.00/0.00 (0.04)
Two	1.24 ** (1.06–1.45)	0.01/0.00 (0.05)
Three or Four	1.40 ** (1.21–1.63)	0.14 **/0.04 (0.05)
Five or More	2.25 ** (1.92–2.58)	0.41 **/0.12 (0.05)
***Covariates***		
Age	0.93 (0.92–0.94)	0.01 **/0.05 (0.00)
Male	1.57 ** (1.42–1.74)	−0.27 **/−0.10 (0.03)
Black	1.64 ** (1.41–1.91)	0.55 **/0.13 (0.05)
Hispanic	1.91 ** (1.60–2.27)	0.51 **/0.13 (0.06)
Asian/Pacific Islander	0.62 ** (0.45–0.85)	0.39 **/0.05 (0.10)
Native American/American Indian	1.78 ** (1.12–2.84)	0.94 **/0.06 (0.16)
Multiracial	1.62 ** (1.24–2.13)	0.35 **/0.04 (0.10)
Other Race/Ethnicity	0.97 (0.60–1.59)	0.00/0.00 (0.16)
Married	0.81 ** (0.71–0.92)	−0.59 **/−0.20 (0.03)
Urbanicity	0.55 ** (0.47–0.63)	−0.13 **/−0.03 (0.04)

Note: ** *p* < 0.01. OR = Odds Ratio. CI = Confidence Interval. B = Unstandardized Coefficient. Beta = Standardized Coefficient. SE = Standard Error. Reference category for categorical measures of ACEs is “zero”. Reference category for Race/Ethnicity is “White”. Estimates are weighted to represent the U.S. population in eligible states of young adults ages 18 to 34 years. Models adjust for state-specific effects. Estimates represent results from the BRFSS 2019 survey.

**Table A6:** Association between adverse childhood experiences and indicators of poor mental health among young adults aged 18–34 (*n* = 14,255).

		*Poor Mental Health Days*	
	*Depression Diagnosis*	*1–13*	*14 or More*
Variables	OR (CI)	RRR (CI)	RRR (CI)
***ACEs***			
One	1.82 ** (1.49–2.23)	1.24 ** (1.07–1.43)	1.70 ** (1.36–2.12)
Two	2.69 ** (2.20–3.30)	1.88 ** (1.60–2.21)	2.99 ** (2.39–3.75)
Three or Four	4.49 ** (3.71–5.43)	2.45 ** (2.10–2.87)	4.66 ** (3.77–5.77)
Five or More	7.69 ** (6.41–9.23)	3.11 ** (2.63–3.68)	9.84 ** (7.97–12.14)
***Covariates***			
Age	1.03 ** (1.02–1.05)	0.97 ** (0.95–0.98)	0.98 * (0.97–0.99)
Male	0.44 ** (0.39–0.50)	0.51 ** (0.46–0.57)	0.45 ** (0.39–0.51)
Black	0.38 ** (0.31–0.46)	0.71 ** (0.60–0.83)	0.61 ** (0.50–0.75)
Hispanic	0.59 ** (0.47–0.73)	0.66 ** (0.55–0.80)	0.68 ** (0.54–0.86)
Asian/Pacific Islander	0.43 ** (0.27–0.68)	0.58 ** (0.42–0.80)	0.58 * (0.38–0.88)
Native American/American Indian	0.86 (0.52–1.41)	0.64 (0.36–1.12)	0.94 (0.52–1.70)
Multiracial	1.01 (0.76–1.35)	1.10 (0.81–1.49)	1.22 (0.85–1.76)
Other Race/Ethnicity	0.44 ** (0.23–0.81)	0.89 (0.50–1.58)	1.03 (0.57–1.87)
Married	0.53 ** (0.46–0.61)	0.76 ** (0.67–0.87)	0.41 ** (0.35–0.49)
Urbanicity	1.19 (0.99–1.42)	1.18 * (1.01–1.39)	1.11 (0.90–1.35)

Note: ** *p* < 0.01; * *p* < 0.05. OR = Odds Ratio. CI = Confidence Interval. RRR = Relative Risk Ratio. Reference Category for Poor Mental Health Days is “0”. Reference category for categorical measures of ACEs is “zero”. Reference category for Race/Ethnicity is “White”. Estimates are weighted to represent the U.S. population in eligible states of young adults ages 18 to 34 years. Models adjust for state-specific effects. Estimates represent results from the BRFSS 2019 survey.
